# Managing complex respiratory patients in the community: an evaluation of a pilot integrated respiratory care service

**DOI:** 10.1136/bmjresp-2016-000145

**Published:** 2016-12-05

**Authors:** K Gillett, K Lippiett, C Astles, J Longstaff, R Orlando, S X Lin, A Powell, C Roberts, A J Chauhan, M Thomas, T M Wilkinson

**Affiliations:** 1 National Institute for Health Research (NIHR) Collaboration for Leadership in Applied Health Research and Care (CLAHRC) Wessex, Respiratory Theme, Southampton, UK; 2 Wessex Academic Health Sciences Network (AHSN), Portsmouth, UK; 3 National Institute for Health Research (NIHR) Collaboration for Leadership in Applied Health Research and Care (CLAHRC) Wessex, Methodological Hub, Southampton, UK; 4 West Hampshire Clinical Commissioning Group (CCG), Eastleigh, UK; 5 Department of Primary Care and Populations Sciences, University of Southampton, Southampton, UK; 6 Department of Clinical and Experimental Sciences, University of Southampton, Southampton, UK

**Keywords:** COPD Exacerbations, Asthma Guidelines, Health Economist

## Abstract

**Introduction:**

In the UK, there is significant variation in respiratory care and outcomes. An integrated approach to the management of high-risk respiratory patients, incorporating specialist and primary care teams' expertise, is the basis for new integrated respiratory services designed to reduce this variation; however, this model needs evaluating.

**Methods:**

To evaluate an integrated service managing high-risk respiratory patients, electronic searches for patients with asthma and chronic obstructive pulmonary disease at risk of poor outcomes were performed in two general practitioner (GP) practices in a local service-development initiative. Patients were reviewed at joint clinics by primary and secondary care professionals. GPs also nominated patients for inclusion. Reviews were delivered to best standards of care including assessments of diagnosis, control, spirometry, self-management, education, medication, inhaler technique and smoking cessation support. Follow-up of routine clinical data collected at 9-months postclinic were compared with seasonally matched 9-months prior to integrated review.

**Results:**

82 patients were identified, 55 attended. 13 (23.6%) had their primary diagnosis changed. In comparison with the seasonally adjusted baseline period, in the 9-month follow-up there was an increase in inhaled corticosteroid prescriptions of 23.3%, a reduction in short-acting β_2_-agonist prescription of 33.3%, a reduction in acute respiratory exacerbations of 67.6%, in unscheduled GP surgery visits of 53.3% and acute respiratory hospital admissions reduced from 3 to 0. Only 4 patients (7.3%) required referral to secondary care. Health economic evaluation showed respiratory-related costs per patient reduced by £231.86.

**Conclusions:**

Patients with respiratory disease in this region at risk of suboptimal outcomes identified proactively and managed by an integrated team improved outcomes without the need for hospital referral.

**Key messages**

- Integrated respiratory clinics delivering joint care by specialists and primary care teams can improve clinical outcomes and reduce care costs for patients with airways disease.

## Introduction

Long-term respiratory conditions in the UK are very common; over 6 million people live with the two most common conditions, asthma and chronic obstructive pulmonary disease (COPD).^1^ Treating respiratory diseases costs the UK National Health Service (NHS) an estimated £4.7 billion annually.^2^ Respiratory disease is the third biggest cause of death in the UK with ∼800 000 patients dying annually.^2^ A high proportion of these costs are generated by a relatively small group of patients with more severe disease or with complex problems that include multimorbidity, at-risk behaviours and socioeconomic disadvantage.^3^ ^4^ These patients often struggle to engage with the structured, proactive care approach to chronic disease management advocated for asthma and COPD, resulting in repeated emergency healthcare use of primary and secondary care.^4^ An integrated approach to the management of complex patients, incorporating specialist and primary care teams' expertise, may be effective in improving outcomes for such high-risk patients. However, the evidence is mixed^5–8^ and there is a need for evaluations of models of integrated care in routine, ‘real-world’ clinical settings.

Over the past two decades, there has been a shift in the locus of care for the majority of patients with chronic respiratory diseases in the UK towards the community.^9^ Respiratory diseases are among the most common causes of primary care consultations, accounting for 24 million consultations annually.^10^ Increasing numbers of complex respiratory patients are being managed in the primary care setting by generalist teams, with a focus on avoidance of admissions to hospital.^9^ Specialist secondary care is restricted to those patients admitted to hospital in a crisis or referred because of uncontrolled disease.^1^ ^10^

There is evidence of significant and unwarranted variability in the standards of respiratory management in the primary and secondary care sectors. Marked variations in outcomes for patients with respiratory disease have also been shown, regionally and between individual general practitioner (GP) practices.^1^ There is evidence linking the quality of care provided in general practice with unplanned admissions to secondary care,^11^ and decreased admission rates have been reported in a number of long-term conditions (including COPD and asthma) where GPs were financially incentivised to provide high-quality care.^12^ Moreover, higher levels of professional education, nurse staffing and clinical recording in primary care are all associated with an improvement in the quality of clinical care for patients with COPD.^13^ However, a ‘skills gap’ may exist in some primary care settings, where GPs and other healthcare professionals lack advanced training in the management of these common conditions, particularly in the case of patients with multimorbidity, uncertain diagnosis or complex problems.^14^ Patients with more severe or complicated disease may receive suboptimal care, which may in turn lead to poor outcomes.^14^ Such patients may fail to reach a specialist assessment that could potentially improve outcomes, either because they are not offered referral to a specialist clinic or because they decline going to a hospital clinic for such an assessment. Therefore, a community-based integrated care approach, harnessing specialist skills and the overall holistic perspective of the generalist primary care teams, is a promising and attractive solution which is being explored by newly commissioned services. Potential benefits of joint specialist–generalist clinics in the community include not only improvement in quality of care for each of the individual patients seen, but also on-site education for the primary care teams, leaving a legacy of improved skills and greater confidence in managing complex disease.^14^ Such clinics may potentially increase patient and staff satisfaction, reduce secondary care use and consequently reduce the financial burden of respiratory disease on the local health economy.^15^

### Local context

The UK region of Wessex is situated on the south coast of England and represents a diverse population of around 2.8 million people, ranging from inner city deprivation to remote rural populations. Local clinical audit data have demonstrated marked variation between local regional administrative groups (eg, a 1.9-fold difference in COPD admission rates and a 2.8-fold difference in asthma admission rates) and between individual GP practices (eg, a 4.7-fold difference in COPD admission rates). Improving respiratory care is an agreed local priority and the basis for newly commissioned integrated respiratory services. West Hampshire Clinical Commissioning Group (CCG), the Wessex Academic Health Sciences Network (WAHSN) and the Wessex Collaboration for Leadership in Applied Health Research and Care (CLAHRC) collaborated to prospectively evaluate a service pilot of an integrated model for managing complex or poorly controlled asthma and COPD across the organisational silos of primary and secondary care with a view to subsequent regional roll-out.

## Methods

### Setting

Two practices were enrolled in the pilot evaluation, one rural and one urban. Patients with poorly controlled COPD and asthma were identified by searches of the practice clinical computer systems, using routinely recorded clinical data. The pilot was registered with the WHCCG as a Quality Improvement project and consultation with the Health Research Authority confirmed the project to qualify as a service evaluation.

### Criteria for patient inclusion and joint-clinic arrangements

COPD patients with poor outcomes were identified using four parameters, those measured in the DOSE Index, a multidimensional assessment tool used to predict outcomes in COPD^16^ whose items are routinely collected in primary care in the UK (Dyspnoea: MRC Breathlessness Score; Obstruction: % predicted FEV1; smoking status; exacerbations). Patients with poorly controlled asthma were identified based on clinical consensus and on at-risk factors identified by the UK National Review of Asthma Deaths (NRAD)^17^ (shown in box 1). Patients were identified through the use of electronic audit and case-finding tools and a manual review of routine patient records by respiratory nurse specialists (RNS). Clinically pertinent information on each patient was prepared in advance of the clinic including the number of exacerbations, hospital admissions and inhaler usage in the 12 months prior to the appointment. Additionally, clinical staff from the GP practice were able to include respiratory patients they considered ‘at risk’ or for whom they required further advice or considered complex.

Box 1 **Inclusion and exclusion criteria for identifying patients for clinics**

Inclusion criteria for identifying patients with complex asthma

- High use of short-acting β_2_-agonist/inhaled corticosteroid combinations (<12 in 12 months).
- High SABA use (>12 in 12 months).
- High SABA use, low ICS use (>12 SABA and <12 ICS in 12 months).
- High oral corticosteroid use (>2 prescriptions for prednisolone in 12 months).
- High doses of ICS monotherapy (≥800 mcg of budesonide (or equivalent)).

Inclusion criteria for identifying patients with complex chronic obstructive pulmonary disease

- Exacerbations within the last 12 months.
- Acute hospital admissions related to respiratory within the last 12 months.
- MRC≥3.
- FEV1<50% predicted.
- At the GP/practice nurse's discretion.

Exclusion criteria

- Pregnancy.
- Housebound.
- Under secondary care for respiratory issue (or other related, eg, cardiac for breathlessness).
- Active cancer.
- At the GP's discretion.

Patients identified were invited to attend for a joint specialist-practice team clinic in the GP premises, including diagnostic review and treatment monitoring from the joint specialist–generalist nursing teams and a clinical assessment from the specialist–generalist medical teams. The practices agreed to provide administrative support which included facilitating electronic searches on their databases, sending out invitations to patients, booking patients into clinics and reminding patients of their appointment date and time, much of which is considered routine clinical practice. There were no financial incentives for the practices. Clinical and information governance arrangements were agreed: the specialist respiratory team was endorsed by the CCG as a guest of the surgeries and the clinical responsibility for the patients remained with the GP. No patient identifiable information was removed from the practice premises.

### The intervention: collaborative clinics

The clinical intervention consisted of a collaborative clinic at the patients' own GP surgery; clinical interventions were driven by clinician–patient interaction rather than predefined protocol. This comprised a 60 min appointment including a 20 min initial nurse assessment, including spirometry and other near-patient diagnostic and monitoring tests appropriate, by a RNS and a practice nurse (PN) or nurse practitioner (NP), followed by a 20 min joint assessment by a respiratory physician (RP) working alongside a practice clinician (GP and/or PN/NP) and a 20 min follow-up education session by an RNS. A personalised disease management and action plan was agreed jointly between the RP, practice clinician and patient. Practical tasks, for example, prescriptions, were carried out by the practice clinician. Relatives and carers were actively encouraged to attend with the patient. The GP retained clinical control and responsibility for the patient.

### Outcome measures

#### Longitudinal follow-up data

Data on exacerbations, medication usage, emergency department (ED)/hospital admissions and GP practice visits were collected from the standard practice clinical electronic records by an RNS 9 months postclinic (February–November 2015). Data were collected through a virtual review of routine medical records for 53 patients who still remained on the GP registers. This was compared to a seasonally matched 9-month period (February–November 2014) prior to clinic.

#### Cost-effectiveness analysis

A cost-effectiveness analysis (CEA) of this project was undertaken: the intervention was the clinic with the outcomes measured in the longitudinal follow-up (postclinic) data; the comparator was the preclinic data. The CEA was performed using standard NHS templates for costing including cost of medication (short-acting β_2_-agonist, SABA and inhaled corticosteroid, ICS),^18^ cost of exacerbation (prescription of antibiotic and oral corticosteroids),^18^ cost of scheduled and unscheduled GP and PN visits,^19^ cost of intervention^19^ (which included clinician and administration time and consumables) and cost of hospital admissions.^20^

#### Statistical analysis: paired non-parametric test

Descriptive statistics have been used in the analysis of the outcome measures. Non-parametric tests (paired Wilcoxon signed-rank tests) have been calculated on the non-parametric data to compare the preclinic and postclinic data after outcome variables display non-normality features; the significance threshold was 0.05.

### Patient and practice feedback

Patients and staff were able to provide unstructured written feedback on the service at the end of their appointment. Feedback was not formally requested from the integrated care team, however a debriefing and education session was held following the completion of the clinics which provided an outlet for feedback from the team members.

## Results

### GP practice demographics

Two GP practices were included in this pilot project. The first was a large, rural market town practice with a practice population of 12 598, staffed by 10 GPs, 3 NPs, 2 PNs and 3 healthcare assistants (HCAs). The practice population deprivation is in the second least deprived decile in the UK; the COPD and asthma prevalence at baseline was 1.8% and 6.7%, respectively (compared to a Wessex average of 1.7% for COPD and 6.3% for asthma). The second was a small, suburban practice with a practice population of 3604 staffed by 1 GP, 1 NP, 2 PNs and 1 HCA. The practice population deprivation is in the fourth least deprived decile in the UK; the baseline COPD and asthma prevalence was 1.7% and 4.6%, respectively.

### Patient demographics

**Figure 1:**
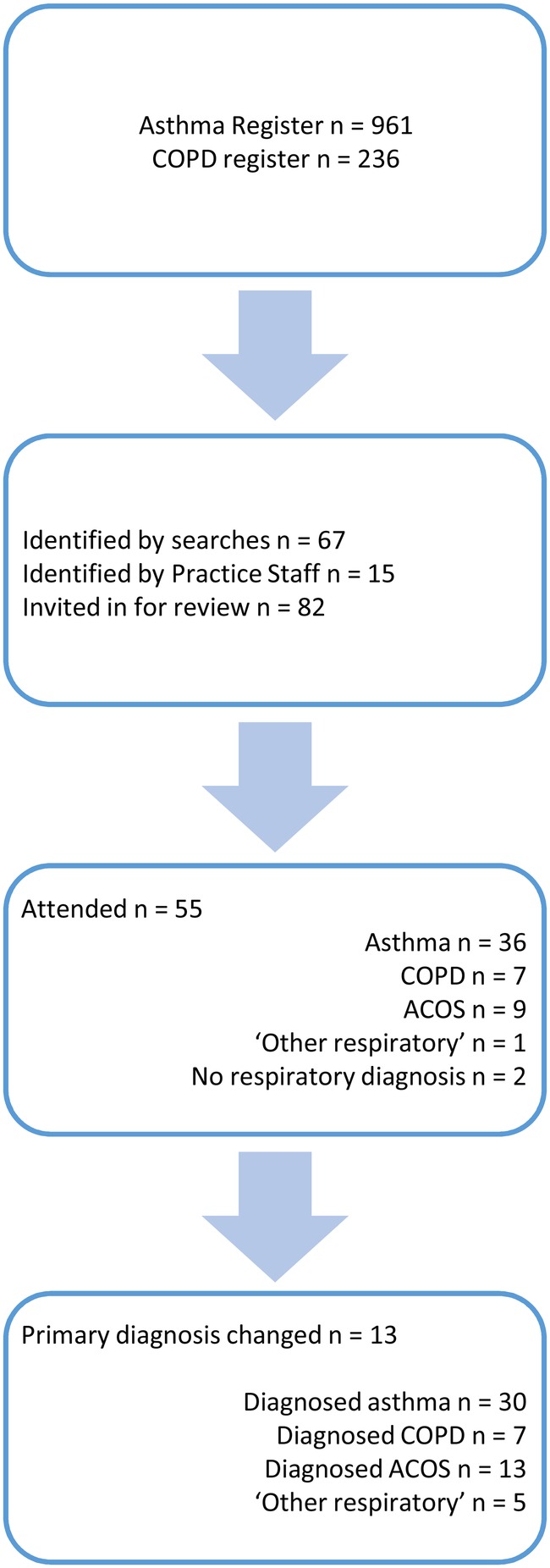
Patient identification, attendance and diagnosis data. ACOS, Asthma and COPD overlap syndrome; COPD, chronic obstructive pulmonary disease.

Eighty-two patients were invited to the intervention clinics, shown in figure 1. Fifty-six of these patients responded and booked an appointment. Of all patients who booked an appointment, 98.2% (55) attended the clinics. Demographics for these patients are shown in table 1.

**Table 1:** Demographics of patients attending clinics

	N (%)
Patients attended	55
Mean age	60
Age range	19–82
Females	33 (60%)
Patients with follow-up data	53
Baseline diagnosis	55
Asthma	36 (65%)
COPD	7 (13%)
Asthma and COPD overlap syndrome (ACOS)	9 (16%)
‘Other respiratory’	1 (2%)
No respiratory diagnosis	2 (4%)
Smoking data	53
Current smoker	11 (21%)
Ex smoker	22 (42%)
Never smoker	20 (38%)
Body mass index (BMI) data	53
BMI <20	0
BMI 20–24	13 (25%
BMI 25–29	20 (38%)
BMI 30–39	18 (24%)
BMI ≥40	2 (4%)

### Clinic data

#### Diagnostic review and accuracy

**Figure 2:**
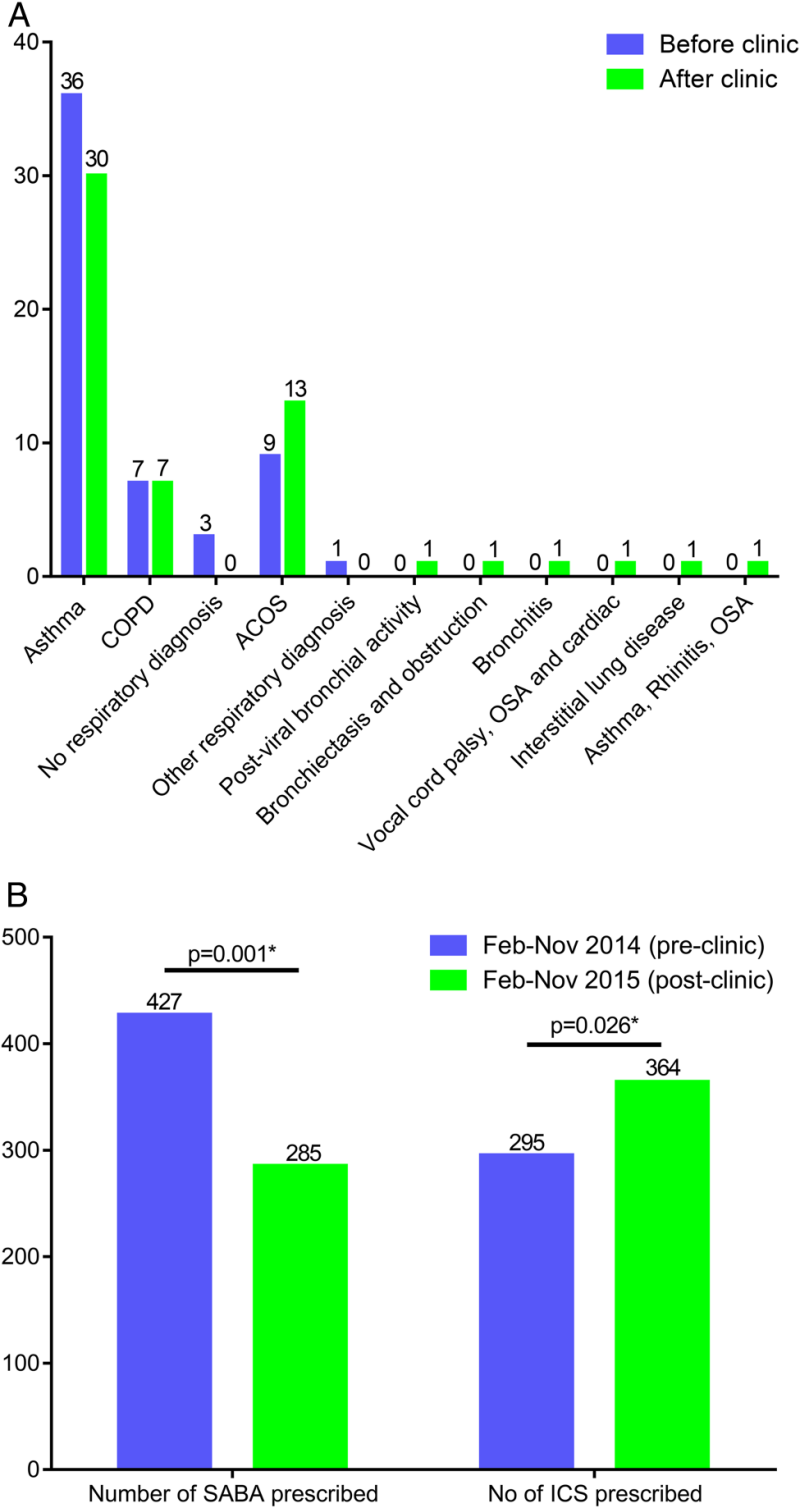
Impacts of clinical review. (A) Summary of changes in clinical diagnosis after clinic review. (B) Summary of inhaled short-acting bronchodilator and inhaled corticosteroid prescription in 9 months prior to and after clinical review. ACOS, Asthma and COPD overlap syndrome; COPD, chronic obstructive pulmonary disease; OSA, obstructive sleep apnoea.

Of the 55 patients seen in the complex clinics, 13 (23.6%) received a change in diagnosis after review by the clinical project team (figure 2A). Reasons for this change in diagnosis included inaccurate initial diagnosis; condition changing over time, for example, asthma to Asthma and COPD overlap syndrome (ACOS); a correct diagnosis had been made previously but not been recorded in the electronic record; the necessary differential diagnostic tests had not been performed to identify the condition.

#### Onward referrals

Of the 55 patients seen, only 4 (7.3%) were referred on to secondary care for further investigations. Thirty-seven (67.3%) required further appointments with their GP/PN for follow-up care such as medication reviews or inhaler technique checks. The remaining 14 (25.4%) did not require any follow-up and returned to the usual routine care with their GP/PN.

## Clinical outcome: longitudinal follow-up data (postclinic).

### Medication usage

#### SABA inhalers

**Figure 3:**
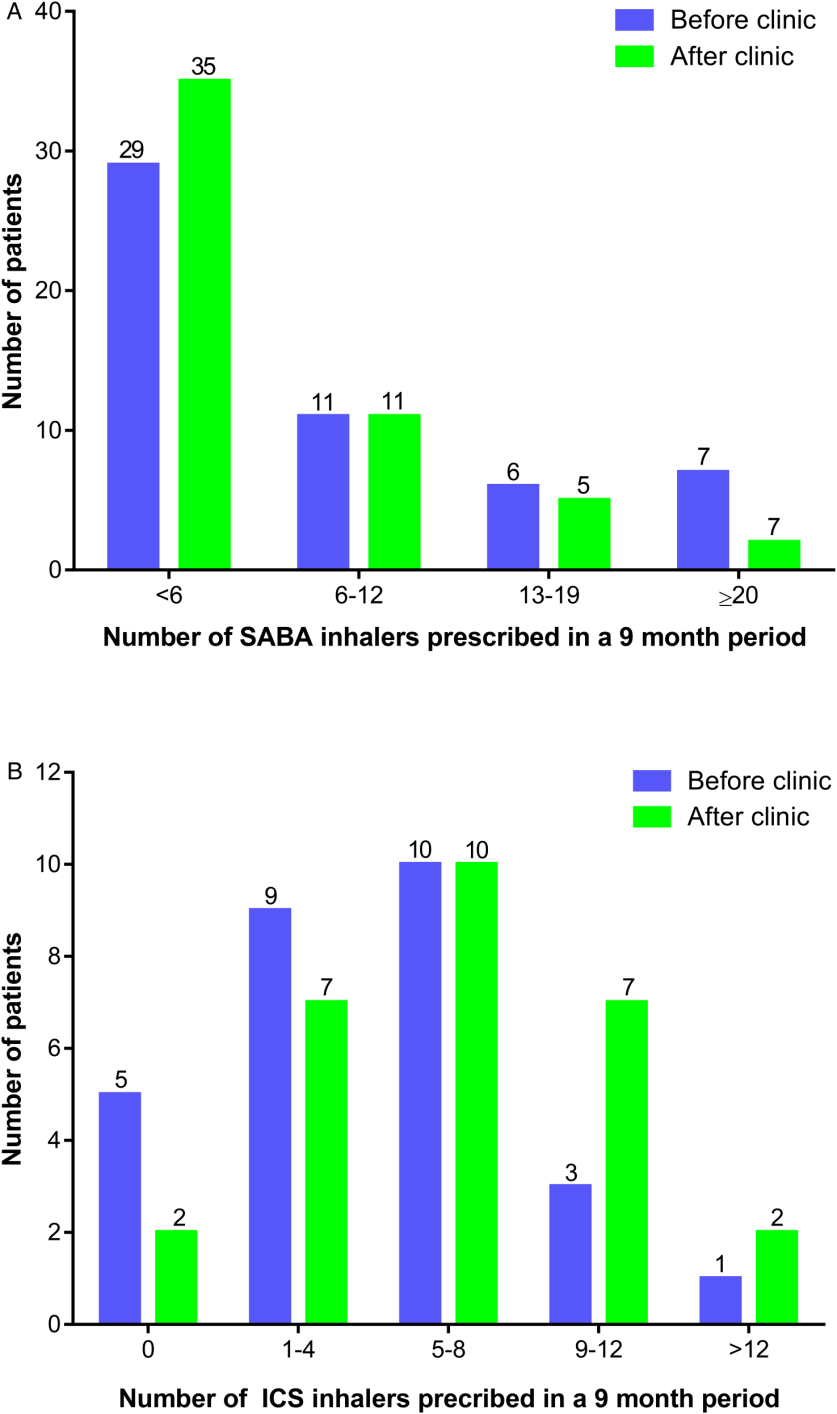
Change in frequency of SABA and ICS prescription after clinical intervention. (A) Frequency of SABA prescriptions preclinic and postclinic (n=53). (B) Frequency of ICS inhaler prescriptions for patients with asthma preclinic and postclinic (n=28).

The number of prescribed SABA inhalers over a 9-month period (February–November 2014) prior to the joint clinic ranged from 0 to 30, with a median of 5. Following the joint clinic, the number of prescribed SABA inhalers ranged from 0 to 26, with a median of 3 over a 9-month period (February–November 2015). The total number of SABA prescribed over the 9-month periods reduced by 33.3% (427 to 285), p<0.001 (see figure 2B). The frequency of prescriptions is shown in figure 3A.

#### ICS inhalers

The total number of ICS-containing inhalers prescribed for all patients increased by 23.3% (295–364), p<0.05 in the 9 months following the specialist clinic compared to the previous seasonally matched 9-month period. A subanalysis of the 28 patients with asthma, in whom ICS adherence is particularly important, was performed, shown in figure 3B. The number of ICS inhalers prescribed over a 9-month period prior to the joint clinic ranged from 0 to 24, with a median of 5. The number of ICS inhalers prescribed over a 9-month period following the joint clinic ranged from 0 to 18, with a median of 6.5.

### Healthcare usage

**Figure 4:**
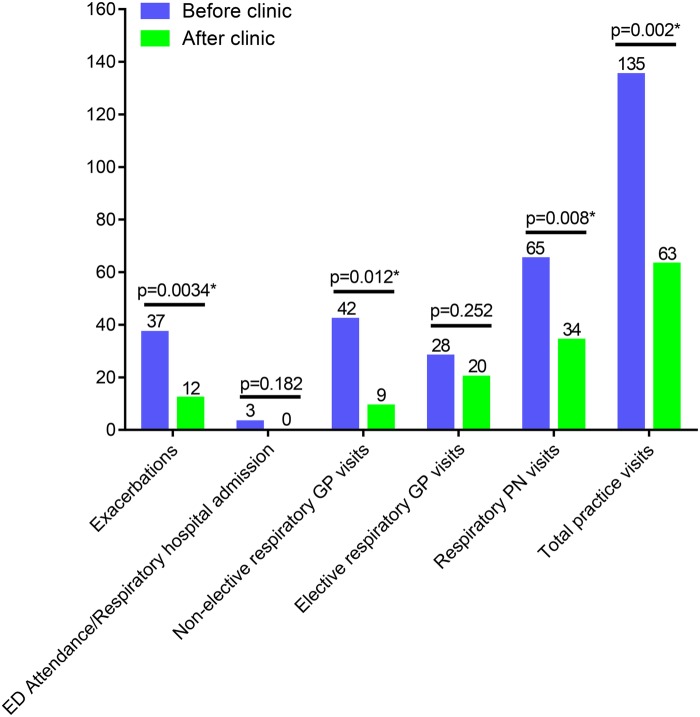
Number of exacerbations, emergency hospital admissions and primary care visits before and after clinics. ED, emergency department; GP, general practitioner.

In the seasonally matched 9-month period preclinic and postclinic, respiratory exacerbations (defined as a non-scheduled contact caused by an acute deterioration in respiratory symptoms resulting in a prescription of oral steroids and/or antibiotics, with a continuation of symptoms without improvement requiring multiple courses of treatment classed as one exacerbation) reduced by 67.6% (from 37 to 12), p<0.01 (figure 4). Non-elective respiratory GP visits (defined as an urgent, unplanned respiratory appointment with the GP) reduced by 78.5% (from 42 to 9), p≤0.01. Elective respiratory GP visits (defined as planned respiratory appointments with the GP for reasons such as medication reviews and clinical review following resolution of an exacerbation) reduced by 28.6% (from 28 to 20), p<0.05. Respiratory PN visits reduced by 47.7% (from 65 to 34), p<0.01. In total, the number of visits to the GP surgery for respiratory issues was reduced by just over half (from 135 to 63), p<0.01.

In the seasonally matched 9-month period preclinic and postclinic, the number of admissions or ED attendances for respiratory issues reduced from 3 to 0, p<0.05. At the time of the follow-up review in November 2015, none of the patients who attended the specialist clinics had died.

### Health economic evaluation

Respiratory-related costs per patient over a 9-month period decrease from £458.11 to £226.25 following the intervention, a reduction of £231.86 per patient, equating to an annualised saving of £309.15 (table 2). The overall cost of the intervention for the 55 patients seen in two practices was £16 325 equating to £296.82 per patient. The incremental cost-effectiveness of the intervention versus no intervention is £142.89 per exacerbation avoided (considered as cost per patient in the timeframe of 9 months).

**Table 2:** Health economic evaluation of clinics: cost per patient over a 9-month period

	Cost preclinic	Cost postclinic
GP visit	£85.27	£35.33
PN visit	£52.00	£27.20
SABA	£29.37	£19.60
ICS	£116.16	£143.32
Exacerbations	£2.44	£0.79
Hospital admissions	£172.88	£0.00
Total cost	£458.11	£226.25

GP, general practitioner; PN, practice nurse; SABA, short-acting β_2_-agonist.

#### Patient feedback

Of the 55 patients who attended, 50 provided unstructured written feedback on the service. All described attending the clinics as a positive experience in terms of patient experience of the clinic and interventions made.

#### Feedback from practices

The informal feedback received from the integrated team members was also extremely positive. Specialist and generalist clinicians found it a useful learning experience with an immediate impact on practice. For example, GPs and PNs stated that they approached inhaler technique training differently after the clinic. RPs mentioned changing the way in which they disseminated information to primary care to include more detailed information.

## Discussion

### Main findings

We present findings from a prospective evaluation of a local service development consisting of an integrated model of care aiming to improve outcomes for complex respiratory patients in the community. The model involved identification of patients with poor outcomes and their review in joint clinics delivered collaboratively by visiting specialist teams and the host primary care teams. This initiative was acceptable to patients and healthcare professionals, including members of the integrated care team. The project demonstrated that informatics approaches to identification of patients with poorly controlled respiratory disease and follow-up is possible and may be conducted using electronic searches of routine clinical data from medical records. Moreover, this intervention can improve both markers of efficient medication use such as improved adherence to preventer inhaled therapy and reduced use of rescue medication and in improved markers of clinical outcomes such as frequency of exacerbation and unscheduled primary care consultations. The health economics analysis demonstrates significant cost savings over usual respiratory care that may cover the additional costs of providing the service, although this will require confirmation in larger studies with a more rigorous health economic evaluation and longer follow-up periods.

The acceptability of the intervention to patients is not only demonstrated through the positive feedback but also by the high response and attendance levels. Some of the patients who attended had repeatedly failed to attend the practice for their routine asthma/COPD annual check-up, a risk factor for exacerbations and premature mortality.^17^ The high level of attendance to the joint respiratory clinics suggests that patients may be more motivated to attend specialist respiratory clinics located in GP surgeries rather than, conventionally, at their local hospital thus potentially providing a more acceptable, efficient and cost-effective service. However, several issues remain—more work is required to identify optimal mechanisms to identify patients with inadequate control and at greatest risk of poor outcome. Furthermore, work is required to address the real unmet need driven by frequent no-attenders to clinical review in routine primary care and in additional services such as this. This population similarly is not involved in current service evaluations and focused research in this group is needed.

The intervention resulted in a change in primary diagnosis for almost a quarter of the patients who attended the clinic. Improving diagnostic accuracy relied particularly on the provision of quality assured spirometry and where needed additional near-patient tests (eg, fraction of exhaled nitric oxide testing). Accurate diagnosis is fundamental to the appropriate provision of preventive therapy,^21^ enabling appropriate management tailored to each condition, for example, ICS in asthma and pulmonary rehabilitation in COPD, and may help to avoid treatment that is of no benefit or harmful.^22^ The shift shown in prescription patterns after clinic review was to one more closely aligned with national guidelines for both COPD. The majority of patients had either a change in diagnosis and/or management made without the need for further input from secondary care, which demonstrates the practicability of this integrated care model in primary care.

Prevention of exacerbations is a key goal in improving control of COPD and asthma.^23^ ^24^ Furthermore, reducing hospital admissions has potential cost savings for the NHS. Emergency hospital admissions for ambulatory care sensitive conditions (ACSC) cost the NHS £1.42 billion annually; 34% of these are for respiratory-related disease.^25^ The results from this project show a reduction in healthcare usage both in terms of exacerbations, hospital admissions and GP surgery attendance. A 68% reduction in exacerbations and 79% reduction in non-elective GP visits are particularly worthy of note and indicate better disease control. The health economic analysis demonstrated that there was a cost saving in terms of healthcare usage following the intervention. Further research with a longer follow-up period is needed to perform an analysis of whether the outcomes from the intervention, for example, reductions in healthcare usage, are sustainable and whether the model will have further cost savings long term as benefits continue to accrue. Further research will also require the collection of quality of life data in order to calculate the cost-effectiveness in terms of cost per quality-adjusted life year (QALY).

This integrated model allowed for shared learning interorganisationally, between primary and secondary care, and intraorganisationally, within the multidisciplinary team. Furthermore, this initiative placed a strong emphasis on the importance of patient and carer education. The emphasis on education for patients, carers and healthcare professionals was intended to leave a legacy of patients who are able to self-manage more effectively and healthcare professionals who are upskilled in the management of patients with respiratory disease and more technically assured in performing specialist respiratory assessments.

### Interpretation of findings in relation to previously published work

This project has demonstrated that this integrated model of care has the potential to improve outcomes for patients with complex respiratory conditions. This is supported by evidence from various NHS organisations including NHS England^26^ and the King's Fund,^14^ which suggest that ensuring specialist support in the delivery of care outside hospital has the potential to improve patient experience and access to care.

### Strengths and limitations

The benefits of this intervention in terms of improving patient outcomes and experience, access to care and reduction in healthcare usage were significant and have the potential to be replicated across the NHS, but require replication in large, well-designed trials. Formal research in a controlled setting (eg, an appropriately powered cluster randomised trial, comparing outcomes in matched practices receiving and not receiving the outreach intervention) is needed.

The intervention in this pilot was a complex and multifactorial one. Consequently, understanding which aspects of the process were key drivers to improvement in an individual is difficult to ascertain; this issue is exacerbated by the relatively small sample size and that patients with asthma and patients with COPD were reviewed. Future larger scale studies and more discrete interventions may be required to tease out relative benefits of diagnostic change from treatment alterations or improved adherence. A more pragmatic approach would be to improve the evidence base behind the improved delivery of guideline adherent care in an integrated team and hence to provide the rationale for commissioners to adopt this model at scale if proven to be cost-effective.

The limited sample size in this pilot restricts the power to detect significant change in hospital admissions, a major driver to costs in respiratory care. However, a reduction was observed, which will again need further investigation in larger controlled trials.

### Implications for future research, policy and practice

Future research in a controlled environment to prove a causal relationship between this integrated respiratory care model and an improvement in respiratory outcomes in a cluster randomised trial is required. If validated this model will then have the potential to be replicated across the NHS and improve respiratory outcomes for patients throughout the UK.

## Conclusions

A range of evidence demonstrates that the current reactive model predicated on acute hospitals is unsustainable in the face of an ageing population, with increasingly complex chronic conditions. This service-development evaluation demonstrates that patients with respiratory disease at risk of suboptimal outcomes can proactively be identified for management by an integrated team in the community without the need for extensive, expensive secondary care technologies and warrants further evaluation at scale to determine its impact in other regions to fully determine health economic outcomes.
